# Validity of algorithms for identifying five chronic conditions in MedicineInsight, an Australian national general practice database

**DOI:** 10.1186/s12913-021-06593-z

**Published:** 2021-06-05

**Authors:** Alys Havard, Jo-Anne Manski-Nankervis, Jill Thistlethwaite, Benjamin Daniels, Rimma Myton, Karen Tu, Kendal Chidwick

**Affiliations:** 1 Alys Havard, NPS MedicineWise, PO Box 1147 , Strawberry Hills, NSW 2012 Sydney, Australia; 2 Medicines Policy Research Unit, Centre for Big Data Research in Health, Faculty of Medicine, UNSW Sydney, Sydney, NSW Australia; 3 Department of General Practice, University of Melbourne, Melbourne, VIC Australia; 4 University of Technology Sydney, Sydney, NSW Australia; 5 Department of Family and Community Medicine, University of Toronto, Toronto, Ontario Canada; 6 North York General Hospital, Toronto, Ontario Canada

**Keywords:** Electronic health records, Primary health care, Chronic disease, Validation study

## Abstract

**Background:**

MedicineInsight is a database containing de-identified electronic health records (EHRs) from over 700 Australian general practices. It is one of the largest and most widely used primary health care EHR databases in Australia. This study examined the validity of algorithms that use information from various fields in the MedicineInsight data to indicate whether patients have specific health conditions. This study examined the validity of MedicineInsight algorithms for five common chronic conditions: anxiety, asthma, depression, osteoporosis and type 2 diabetes.

**Methods:**

Patients’ disease status according to MedicineInsight algorithms was benchmarked against the recording of diagnoses in the original EHRs. Fifty general practices contributing data to MedicineInsight met the eligibility criteria regarding patient load and location. Five were randomly selected and four agreed to participate. Within each practice, 250 patients aged ≥ 40 years were randomly selected from the MedicineInsight database. Trained staff reviewed the original EHR for as many of the selected patients as possible within the time available for data collection in each practice.

**Results:**

A total of 475 patients were included in the analysis. All the evaluated MedicineInsight algorithms had excellent specificity, positive predictive value, and negative predictive value (above 0.9) when benchmarked against the recording of diagnoses in the original EHR. The asthma and osteoporosis algorithms also had excellent sensitivity, while the algorithms for anxiety, depression and type 2 diabetes yielded sensitivities of 0.85, 0.89 and 0.89 respectively.

**Conclusions:**

The MedicineInsight algorithms for asthma and osteoporosis have excellent accuracy and the algorithms for anxiety, depression and type 2 diabetes have good accuracy. This study provides support for the use of these algorithms when using MedicineInsight data for primary health care quality improvement activities, research and health system policymaking and planning.

**Supplementary Information:**

The online version contains supplementary material available at 10.1186/s12913-021-06593-z.

## Background

Electronic health records (EHRs) are used in primary health care settings to keep patient-level records of clinical information including diagnoses, reasons for encounters, prescriptions, observations, test results and referrals [1]. The development of tools to extract the data contained in these EHRs has allowed for the establishment of primary health care EHR databases which have proven to be a valuable resource for health research and public health surveillance. Widely used examples from across the world include the Clinical Practice Research Datalink (CPRD) [2] and The Health Improvement Network (THIN) database [3] in the United Kingdom (UK), and the Canadian Primary Care Sentinel Surveillance Network (CPCSSN) [4]. Primary health care EHR data have been used to improve our understanding of the epidemiology of diseases and the use, costs and outcomes of health care practices, as well as for disease surveillance and quality improvement in primary health care [2, 5, 6].

In Australia, the majority of general practitioners use EHRs to manage their patient care, including writing prescriptions, ordering pathology tests and filing correspondence. A variety of EHR clinical information systems are in use, all with different data structures and terminologies. This lack of interoperability means that EHR data is not routinely shared between practices, although efforts to change this are underway with the introduction of the national cloud-based My Health Record [7]. There has been limited use of Australian primary health care EHR data for research and surveillance, because of data access barriers [1, 8]. These obstacles have been overcome by the establishment of centralised repositories, which are now facilitating timely access to EHR data from Australian general practices [8]. MedicineInsight, which has national coverage, is one of the largest and most widely used of these Australian databases. Details of this resource are described elsewhere [9]. Briefly, MedicineInsight was established in 2011 and contains de-identified EHRs from just over 700 of Australia’s 8147 general practices [10]. MedicineInsight focuses on practices using Best Practice [BP]^™^ or Medical Director [MD]^™^, the most widely used clinical information systems in Australia (over 80 % coverage) and the most similar in structure, noting that they were designed by the same individual [7]. A whole-of-practice data collection, containing all available EHRs in the practice’s clinical information system is conducted when a practice joins MedicineInsight. Extracted data include patient demographics and clinical data entered directly into fields within the EHR by healthcare professionals. Free text fields potentially containing identifying information, such as progress notes and correspondence, are not included in the extraction. Incremental data are extracted regularly, resulting in an updated longitudinal database in which patients within each practice can be tracked over time. Data from practices using BP and MD software are merged into a single consistent data structure, and monthly builds of the database are generated and made available for use.

As is the case for many of these primary health care EHR databases, MedicineInsight contains diagnostic algorithms [1] that use information from various EHR fields to identify whether patients have specific health conditions. Such algorithms are required because there is no single field that provides definitive information on the health conditions experienced by each patient. The MedicineInsight algorithms have been developed by NPS MedicineWise, the custodian of MedicineInsight, to create efficiencies for users of the data and promote consistency between studies.

Knowledge of the extent to which these algorithms accurately identify patients’ disease status is key to understanding the potential biases that may arise in analyses using these algorithms. This is essential for the appropriate interpretation of results of analyses of MedicineInsight data. Indeed, validation studies of algorithms used to identify patients with health conditions in routinely collected data have been recognized as a priority for health services research [11, 12]. Although the MedicineInsight algorithms for many conditions have been demonstrated to yield prevalence estimates that are similar to those produced by other reputable data sources [13–15], there has been no formal assessment of their validity. The findings from the numerous validation studies of diagnostic algorithms in primary health care EHR data in other developed countries [16] cannot be assumed to generalise to Australian data, due to between-country differences in the operation and funding of the health care system and differences in the variables available in different databases [12].

The purpose of this study was to examine the validity of MedicineInsight algorithms for five common chronic conditions in general practice: anxiety, asthma, depression, osteoporosis and type 2 diabetes.

## Methods

 We compared each patient’s disease status according to the diagnostic algorithms in the MedicineInsight database to their status determined through review of the original EHRs held in the participating practices.

### Study population

This study was based on patients attending four general practices participating in MedicineInsight. To be eligible, practices had to meet the following criteria:

i) data related to activity in October 2019 were successfully extracted;
ii) at least 250 patients aged 40 years and older with an encounter in October 2019;
iii) located within 40 km of the Sydney or Melbourne central business districts, to ensure ease of access for EHR reviewers (Sydney and Melbourne are the capital cities of Australia’s two most populous states); and.
iv) participated in at least one MedicineInsight quality improvement activity in the period November 2018 to October 2019, to ensure interest in engaging with the MedicineInsight program.

We categorised the 50 practices meeting these criteria according to the EHR software used (BP or MD) and the city in which the practice is located (Sydney or Melbourne). We randomly selected one practice from each of these four categories (BP Sydney, MD Sydney, BP Melbourne and MD Melbourne); additional practices were selected until one from each category agreed to participate. We stratified our random selection by the EHR software used so that we could examine whether the software contributed to any differences in the validity of the MedicineInsight algorithms. We stratified by city to evenly distribute the data collection between EHR reviewers based in the two cities. Five practices were issued with invitations to participate before four confirmed participation by providing written informed consent.

Using MedicineInsight data, we selected patients who were aged 40 years and older and attended the participating practices in October 2019. This age restriction increased the prevalence of the evaluated conditions, thereby optimising statistical power. We randomly selected 250 of these patients per practice. We aimed to collect data for as many of these patients as possible within the five days of data collection planned at each practice.

### MedicineInsight diagnostic algorithms

MedicineInsight personnel have developed coding algorithms that identify patients with specific health conditions. These algorithms identify conditions using information from three EHR fields: *diagnosis, reason for visit* and *reason for prescription*. These fields either contain coded terms that the user selects from a drop-down list in the EHR software, or free text. ‘Pyefinch’ coding is available in BP, while ‘Docle’ coding is available in MD. The algorithms identify patients as having the specific health condition if a coded term or text string from the pre-defined list has ever been recorded for that patient in any one of the three fields. The pre-defined list of coded terms and text strings is compiled by trained clinical coders, and is based on available Pyefinch and Docle codes, as well as commonly accepted clinical definitions and abbreviations. For records identified by a free text string alone, the context in which it is recorded is reviewed by clinical coders at the time of developing the algorithm and periodically thereafter, and irrelevant instances removed. A detailed description of the MedicineInsight algorithms for anxiety, asthma, depression, osteoporosis and type 2 diabetes is included in Additional File 1.

For the purposes of this study, the diagnostic algorithms were applied to MedicineInsight data up to 31 October 2019. To ensure that the results of EHR reviews could not influence the classification of patients on the diagnostic algorithms, values on the diagnostic algorithms were extracted from the MedicineInsight database prior to the conduct of EHR reviews. These data extracts were provided to an analyst who did not have access to any additional MedicineInsight data.

### EHR reviews

Information obtained from the original EHRs held in the participating practices was used as the reference standard against which accuracy was benchmarked. Three EHR reviewers visited the participating practices between January and March 2020 and accessed the original EHRs for the randomly selected patients. All EHR reviewers were health professionals registered with the Australian Health Practitioner Regulation Agency, and thus accredited for the keeping of medical records and adherence to confidentiality and privacy principles. Anonymised identifiers for these patients (extracted from the MedicineInsight data) were reassociated with patient names using the third-party data extraction tools installed on computers at each practice. EHR reviewers completed reviews for as many of the 250 selected patients as possible within the time available in the practice, which ranged from three to eight days. To minimise the inconvenience to practices, we planned only five days of data collection in each practice. In one practice, EHR reviews were particularly time consuming due to the size of the records, so an extra three days of data collection were completed. In two of the practices, it was necessary to close data collection early due to COVID-19, with three days of data collection completed in one, and four days in the other. EHR reviewers worked through the randomly ordered list of selected patients from the beginning, without skipping any.

Guided by a standardised electronic data capture form, the EHR reviewers searched for evidence of the specific health conditions in the following fields: *diagnosis, reason for visit, reason for prescription, correspondence* and *progress notes*. If a diagnosis of the condition (recording of symptoms was not sufficient) was recorded in any of these fields, or if it was documented that the patient was undergoing treatment that is highly specific to the specific condition (e.g. asthma care plan), the patient was considered to have the condition. The term ‘anxiety’ was the exception; it can be used to represent symptoms, but it is often used to indicate anxiety disorder. If it was not clear from the context whether the term ‘anxiety’ was meant to represent symptoms or a diagnosis, it was assumed to be a diagnosis. For osteoporosis, the *investigations/results* fields were also searched for a diagnosis recorded on bone mineral density test results. The *investigations/results* fields were also searched for type 2 diabetes. If a diagnosis was recorded or results of fasting blood glucose tests, oral glucose tolerance tests or glycated haemoglobin tests were consistent with the Royal Australian College of General Practitioners’ diagnostic criteria for type 2 diabetes [17], the patient was considered to have type 2 diabetes. EHR reviewers were blinded to the patient’s disease status on the MedicineInsight algorithms. EHR reviewers were instructed to ignore any evidence documented after 31 October 2019, as the algorithms were applied to MedicineInsight data up to this date. The EHR data were collected and managed using REDCap electronic data capture tools hosted at The University of Melbourne.

### Analysis

For each health condition, the sensitivity, specificity, positive predictive value (PPV) and negative predictive value (NPV) of the MedicineInsight algorithms were calculated. These measures of accuracy are defined in Table 1. As the data are clustered within practices, variance was adjusted to account for correlation between observations within clusters, and confidence intervals adjusted accordingly. Analyses were conducted using R, version 3.6.2 [18].

**Table 1:** Definitions of measures of accuracy

Sensitivity: the number of patients that are identified by the MedicineInsight algorithm as having the specific health condition, as a proportion of patients who truly have the specific health condition (according to EHR reviews)
**Specificity**: the number of patients that are identified by the MedicineInsight algorithm as not having the specific health condition, as a proportion of patients who truly do not have the specific health condition
**Positive predictive value (PPV)**: the number of patients that truly have the specific health condition, as a proportion of patients identified by the MedicineInsight algorithm as having the specific health condition.
**Negative predictive value (NPV)**: the number of patients that truly do not have the specific health condition, as a proportion of patients identified by the MedicineInsight algorithm as not having the specific health condition.

## Results

Within the time available for data collection, EHR reviews were conducted for 477 patient records. One of these EHR reviews was not included in the analyses because the EHR indicated that it was a test record (as opposed to belonging to a real patient), while another was excluded because the EHR review record could not be linked to a patient record in the MedicineInsight data extract due to a data entry error in the study patient identifier. This resulted in the inclusion of 475 patients in the analysis, distributed across practices as follows: BP Sydney, 3 days, *n* = 65 (14 %); MD Sydney, 5 days, *n* = 194 (41 %); BP Melbourne, 8 days, *n* = 110 (23 %); and MD Melbourne, 4 days, *n* = 106 (22 %). 40 % of the final sample were male; 61 % were aged 40 to 64 years, with the remainder 65 years or older; and 37 % had EHRs based on BP software, with the remainder in MD software.

Concordance between the MedicineInsight diagnostic algorithms and EHR reviews is presented in Table 2. Based on EHR reviews for these 475 patients aged ≥ 40 years, 163 (34 %) patients had anxiety. The diagnostic algorithm for identifying patients with anxiety yielded excellent specificity, PPV and NPV (all 0.93 and above) and a sensitivity of 0.85. According to EHR reviews, 23 % of patients had a diagnosis of asthma recorded ever, and 11 % had osteoporosis. The diagnostic algorithms for asthma and osteoporosis both yielded excellent sensitivity, specificity, PPV and NPV (all 0.94 and above). 35 % of patients ever had a diagnosis of depression recorded, and 15 % had type 2 diabetes. The diagnostic algorithms for depression and type 2 diabetes yielded excellent specificity, PPV and NPV (all 0.94 and above), and both yielded a sensitivity of 0.89.

**Table 2:** Concordance between the MedicineInsight diagnostic algorithms and EHR reviews for five chronic conditions

	EHR+ Algorithm+ (True positive)	EHR– Algorithm+ (False positive)	EHR+ Algorithm- (False negative)	EHR- Algorithm- (True negative)	Sensitivity (95 % CI)	Specificity (95 % CI)	PPV (95 %CI)	NPV (95 % CI)
**Anxiety**	139	7	24	305	0.85 (0.77–0.91)	0.98 (0.96–0.99)	0.95 (0.90–0.98)	0.93 (0.87–0.96)
**Asthma**	98	1	10	366	0.91 (0.79–0.96)	1.00 (0.98–1.00)	0.99 (0.93–1.00)	0.97 (0.92–0.99)
**Depression**	149	7	19	300	0.89 (0.86–0.91)	0.98 (0.96–0.99)	0.96 (0.92–0.98)	0.94 (0.92–0.96)
**Osteoporosis**	48	2	3	422	0.94 (0.86–0.98)	1.00 (0.98–1.00)	0.96 (0.83–0.99)	0.99 (0.99–1.00)
**Type 2 diabetes**	63	2	8	402	0.89 (0.83–0.93)	1.00 (0.98–1.00)	0.97 (0.92–0.99)	0.98 (0.97–0.99)

When the calculation of these measures of accuracy was stratified according to the EHR software used (BP or MD), non-overlapping confidence intervals indicated statistically significant differences in the NPV for asthma (0.93, 95 % CI: 0.90–0.95 in BP and 1.00, 95 % CI: 0.99–1.00 in MD), the PPV for osteoporosis (1.00, 95 % CI: 0.98–1.00 in BP and 0.92, 95 % CI: 0.80–0.97 in MD), and the specificity for type 2 diabetes (0.99, 95 % CI: 0.98–0.99 in BP and 1.00, 95 % CI: 1.00–1.00 in MD). While statistically significant, these differences have no obvious clinical significance (see Table 3).

**Table 3:** Concordance between the MedicineInsight diagnostic algorithms and EHR reviews for five chronic conditions, stratified by EHR software

		EHR+ Algorithm+ (True positive)	EHR– Algorithm+ (False positive)	EHR+ Algorithm- (False negative)	EHR- Algorithm- (True negative)	Sensitivity (95 % CI)	Specificity (95 % CI)	PPV (95 %CI)	NPV (95 % CI)
Best Practice	**Anxiety**	49	1	13	112	0.79 (0.77–0.81)	0.99 (0.97–1.00)	0.98 (0.95–0.99)	0.90 (0.87–0.92)
	**Asthma**	47	1	9	118	0.84 (0.67–0.93)	0.99 (0.96–1.00)	0.98 (0.84–1.00)	0.93 (0.90–0.95)
	**Depression**	55	1	6	113	0.90 (0.85–0.94)	0.99 (0.95–1.00)	0.98 (0.90–1.00)	0.95 (0.91–0.97)
	**Osteoporosis**	25	0	1	149	0.96 (0.89–0.99)	1.00 (1.00–1.00)	1.00 (0.98–1.00)	0.99 (0.98–1.00)
	**Type 2 diabetes**	37	2	3	133	0.92 (0.84–0.97)	0.99 (0.98–0.99)	0.95 (0.91–0.97)	0.98 (0.94–0.99)
Medical Director	**Anxiety**	90	6	11	193	0.89 (0.81–0.94)	0.97 (0.95–0.98)	0.94 (0.89–0.97)	0.95 (0.88–0.98)
	**Asthma**	51	0	1	248	0.98 (0.93–1.00)	1.00 (1.00–1.00)	1.00 (0.93–1.00)	1.00 (0.99–1.00)
	**Depression**	94	6	13	187	0.88 (0.85–0.91)	0.97 (0.95–0.98)	0.94 (0.91–0.96)	0.94 (0.91–0.95)
	**Osteoporosis**	23	2	2	273	0.92 (0.80–0.97)	0.99 (0.98–1.00)	0.92 (0.80–0.97)	0.99 (0.98–1.00)
	**Type 2 diabetes**	26	0	5	269	0.84 (0.66–0.95)	1.00 (1.00–1.00)	1.00 (0.87–1.00)	0.98 (0.97–0.99)

## Discussion

This study found that all five MedicineInsight diagnostic algorithms evaluated had excellent specificity, PPV and NPV. The high specificities and PPVs indicate that these algorithms return few false positives and are therefore useful for identifying cohorts of patients who truly have the specific condition and for classifying outcomes [19]. The asthma and osteoporosis algorithms also had excellent sensitivity, making them valuable for identifying representative cohorts of patients and for measuring the prevalence of these conditions. The algorithms for anxiety, depression and type 2 diabetes yielded sensitivities below 0.9, which indicates that some patients who have these conditions are incorrectly classified as not having these conditions. As a result, use of these algorithms will lead to undercounting of patients with these conditions and this should be borne in mind when interpreting the findings of analyses involving these algorithms. Nevertheless, this level of under ascertainment is generally considered acceptable, with many prior validation studies of primary health care EHR data interpreting sensitivities of this magnitude as evidence of good accuracy [4, 16, 20].

Three of the evaluated MedicineInsight diagnostic algorithms have accuracy that is comparable to, or superior to, the accuracy of diagnostic algorithms in electronic primary health care databases in other parts of the world. According to a recent systematic review, other asthma algorithms have yielded sensitivities ranging from 0.74 to 0.92, specificities ranging from 0.84 to 0.98, PPVs ranging from 0.67 to 0.81 and NPVs of 0.9 and above [16]. Depression algorithms have returned sensitivities ranging from 0.73 to 0.81, PPVs ranging from 0.79 to 0.87 and specificities and NPVs of 0.9 and above [16]. Type 2 diabetes algorithms have yielded sensitivities ranging from 0.65 to 1.0, PPVs ranging from 0.87 to 1.0 and specificities and NPVs of 0.94 and above [16]. To our knowledge, there have been no prior validation studies of anxiety or osteoporosis algorithms in primary health care data.

### Strengths and limitations

A strength of this study is that EHR reviews were conducted for patients that the algorithm identified as cases as well as those the algorithm considered non-cases. Including both cases and non-cases in a study allows for the calculation of sensitivity, specificity, NPV and PPV, where all of these measures are important because each describes a different aspect of accuracy and allows the reader to consider how the algorithm will perform in a particular context [19]. Despite this, many studies have not collected reference standard data for non-cases, instead opting to seek confirmation only for patients identified as cases by the algorithm. While this reduces the total number of patients for whom reference standard data needs to be collected, such an approach means that PPV is the only measure of accuracy that can be calculated. To attain sufficient statistical power in the current study, the sample was restricted to patients aged 40 years and older. This represents a trade-off in terms of generalisability of the PPV and NPV estimates. As estimates of PPV and NPV depend on the prevalence of the specific health condition [11], the PPV estimates returned in this study may be higher, and our NPV estimates may be lower, than those yielded by the diagnostic algorithms in a population with a lower prevalence of the condition. The prevalence of the five conditions in our sample was approximately twice that of the whole MedicineInsight patient sample [14]. In addition to the age restriction, this increased prevalence is likely due to the focus on patients with a recent visit to a general practitioner, the chance of which would be higher in frequent attenders. A further threat to the generalisability of the results arises from the inclusion of only four practices in this study, potentially leading to high sampling variability, compounded by the uneven distribution of EHR reviews across these practices. As a consequence, in the estimates of concordance generated by this study, more weight has been given to those practices that contributed more EHR reviews. This uncertain generalisability should be borne in mind when applying the diagnostic algorithms for other populations within the MedicineInsight database.

Recording of the diagnosis in the original EHRs was used as the reference standard against which the accuracy of the algorithms was benchmarked. The limitation of this approach is that the recording of diagnoses in the original EHR may be inaccurate or incomplete [19]. This is a particular challenge in the Australian context, where patients are able to obtain care at multiple general practices and information is not routinely shared between practices. The extent to which diagnoses are not recorded completely may differ according to the specific condition, with fragmentation of mental health care and patient concerns about confidentiality contributing to the under-recording of mental health conditions in primary health care EHRs [21]. Despite this, there is consensus among experts that EHR reviews are an acceptable reference standard for validation studies, with the majority of validation studies of electronic primary health care and other administrative health data using EHR reviews as the reference standard [11, 16]. As an alternative to EHR reviews, some prior validation studies have asked general practitioners to complete questionnaires regarding the health of their individual patients. However, this approach generally results in a low response rate and limits the number of patients for whom data can be collected [22]. Other validation studies have used records in population-based data collections such as cancer registries, hospital admissions data and death registries as the reference standard [23, 24], but this is not possible for MedicineInsight data until full-scale record linkage is enabled.

## Conclusions

Primary health care EHR databases are powerful resources for improving our understanding of health and healthcare practices. These databases typically provide clinical information that is richer than that available through administrative data or population surveys [1]. However, the extent to which the findings of analyses of such data are a true reflection of patient health, and are trusted by clinicians, policymakers and researchers, depends on the accuracy of the data. This study measured the accuracy of MedicineInsight algorithms for five chronic conditions, finding that the algorithms for asthma and osteoporosis have excellent accuracy and the algorithms for anxiety, depression and type 2 diabetes have good accuracy when compared to recording of diagnoses in the original EHR. This study provides support for the use of these algorithms in the MedicineInsight data for primary health care quality improvement activities, research and health system policymaking and planning.

 General practices provided informed written consent to participate in this research, and a waiver of the requirement for individual patient consent was granted by the NREEC.

## Supplementary Information

**Additional file 1:**

## Data Availability

The data used in this study are not publicly available due to the risk to individual patients’ confidentiality and privacy. However, other researchers may be able to access the data used in this study provided approval is granted by the MedicineInsight Data Governance Committee and the Royal Australian College of General Practitioners (RACGP) National Research and Evaluation Ethics Committee. Data access enquiries can be directed to NPS MedicineWise (medicineinsight@nps.org.au).

## References

[1] Youens D, Moorin R, Harrison A, Varhold R, Robinson S, Brooks C (2020). Using general practice clinical information system data for research: the case in Australia. Int J Popul Data Sci.

[2] Herrett E, Gallagher A, Bhaskaran K, Forbes H, Mathur R, van Staa T (2015). Data Resource Profile: Clinical Practice Research Datalink (CPRD). Int J Epidemiol.

[3] Blak B, Thompson M, Dattani H, Bourke A (2011). Generalisability of The Health Improvement Network (THIN) database: demographics, chronic disease prevalence and mortality rates. Inform Prim Care.

[4] Williamson T, Green M, Birtwhistle R, Khan S, Garies S, Wong S (2014). Validating the 8 CPCSSN case definitions for chronic disease surveillance in a primary care database of electronic health records. Ann Fam Med.

[5] Greiver M, Drummond N, Birtwhistle R, Queenan J, Lambert-Lanning A, Jackson D (2015). Using EMRs to fuel quality improvement. Can Fam Physician.

[6] Mannan F, Chaudhry Z, Gibson-White A, Syed U, Ahmed S, Kousoulis A (2017). Outputs and growth of primary care databases in the United Kingdom: bibliometric analysis. J Innov Health Inform.

[7] Gordon J, Miller G, Britt H. Reality check - reliable national data from general practice electronic health records. Deeble Institute Issue Brief No. 18: Deeble Institute; 2016 [Available from: https://ahha.asn.au/system/files/docs/publications/deeble_institue_issues_brief_no_18.pdf.

[8] Canaway R, Boyle D, Manski-Nankervis J, Bell J, Hocking J, Clarke K (2019). Gathering data for decisions: best practice use of primary care electronic records for research. Med J Aust.

[9] Busingye D, Gianacas C, Pollak A, Chidwick K, Merrifield A, Norman S (2019). Data Resource Profile: MedicineInsight, an Australian national primary health care database. Int J Epidemiol.

[10] Australian Government Productivity Commission. Report on Government Services 2021, Part E, Secs. 10, Table 10A.53 2021 [Available from: https://www.pc.gov.au/research/ongoing/report-on-government-services/2021/health/primary-and-community-health.

[11] Benchimol E, Manuel D, To T, Griffiths A, Rabeneck L, Guttmann A (2011). Development and use of reporting guidelines for assessing the quality of validation studies of health administrative data. J Clin Epidemiol.

[12] Ehrenstein V, Petersen I, Smeeth L, Jick S, Benchimol E, Ludvigsson J (2016). Helping everyone do better: a call for validation studies of routinely recorded health data. Clinical Epidemiology.

[13] Heron J, Norman S, Yoo J, Lembke K, O’Connor C, Weston C (2019). The prevalence and risk of non-infectious comorbidities in HIV-infected and non-HIV infected men attending general practice in Australia. PLoS One.

[14] NPS MedicineWise (2020). General Practice Insights Report July 2018-June 2019.

[15] Gonzalez-Chica D, Vanlint S, Hoon E, Stocks N (2018). Epidemiology of arthritis, chronic back pain, gout, osteoporosis, spondyloarthropathies and rheumatoid arthritis among 1.5 million patients in Australian general practice: NPS MedicineWise MedicineInsight dataset. BMC Musculoskelet Disord.

[16] McBrien K, Souri S, Symonds N, Rouhi A, Lethebe B, Williamson T (2018). Identification of validated case definitions for medical conditions used in primary care electronic medical record databases: a systematic review. J Am Med Inform Assoc.

[17] The Royal Australian College of General Practitioners (2016). General practice management of type 2 diabetes: 2016–2018.

[18] R Core Team. R: A Language and Environment for Statistical Computing Vienna, Austria: R Foundation for Statistical Computing; 2019 [cited 10 June 2020]. Available from: https://www.R-project.org.

[19] Chubak J, Pocobelli G, Weiss N (2012). Trade-offs between accuracy measures for electronic healthcare data algorithms. J Clin Epidemiol.

[20] Kadhim-Saleh A, Green M (2013). Validation of the diagnostic algorithms for 5 chronic conditions in the Canadian Primary Care Sentinel Surveillance Network (CPCSSN): A Kingston Practice-based Research Network (PBRN) Report. J Am Board Fam Med.

[21] Madden J, Lakoma M, Rusinak D, Lu C, Soumerai S (2016). Missing clinical and behavioural health data in a large electronic health record (EHR) system. J Am Med Inform Assoc.

[22] Herrett E, Thomas S, Schoonen W, Smeeth L, Hall A (2009). Validation and validity of diagnoses in the General Practice Research Database: a systematic review. Br J Clin Pharmacol.

[23] Dregan A, Moller H, Murray-Thomas T, Gulliford M (2012). Validity of cancer diagnosis in a primary care database compared with linked cancer registrations in England: Population-based cohort study. Cancer Epidemiol.

[24] Thomas K, Davies N, Metcalfe C, Windmeijer F, Martin R, Gunnell D (2012). Validation of suicide and self-harm records in the Clinical Practice Research Datalink. Br J Clin Pharmacol.

