# Supplementary material for: Validity of algorithms for identifying five chronic conditions in MedicineInsight, an Australian national general practice database
Source: BMC Health Serv Res. 2021 Jun 5;21:551. doi: 10.1186/s12913-021-06593-z (PMC8178900; doi:10.1186/s12913-021-06593-z)
Supplement: Supplementary file 1 — Additional file 1: [file 12913_2021_6593_MOESM1_ESM.docx]

**Validity of algorithms for identifying five chronic conditions in MedicineInsight, an Australian national general practice database**

Alys Havard^1,2^, Jo-Anne Manski-Nankervis^3^, Jill Thistlethwaite^1,4^, Benjamin Daniels^1,2^, Rimma Myton^1^, Karen Tu^5,6^, Kendal Chidwick^1^

^1^ NPS MedicineWise, Sydney, NSW, Australia

^2^ Medicines Policy Research Unit, Centre for Big Data Research in Health, Faculty of Medicine, UNSW Sydney, Sydney, NSW, Australia

^3^ Department of General Practice, University of Melbourne, Melbourne, VIC, Australia

^4^ University of Technology Sydney, Sydney, NSW, Australia

^5^ Department of Family and Community Medicine, University of Toronto, Toronto, Ontario, Canada

^6^ North York General Hospital, Department of Family and Community Medicine, University of Toronto, Toronto Western Hospital Family Health Team-University Health Network, Toronto, Ontario, Canada

**Corresponding author:** Alys Havard, NPS MedicineWise, PO Box 1147 Strawberry Hills, NSW 2012, Australia. [ahavard@nps.org.au](mailto:ahavard@nps.org.au)

# additional file 1: Description of the medicineinsight algorithms evaluated

## Anxiety

This flag indicates those records where Anxiety (or a relevant synonym) is reported in MedicineInsight either as a coded condition (using a drop-down list in the CIS) or as a non-coded condition (free text) in one or more of the 'Diagnosis', 'Reason for visit' or 'Reason for prescription' fields.

Note: This flag reflects Anxiety broadly understood and includes:

- anxiety
- generalised anxiety disorder
- social anxiety disorder

It does not include (when documented in isolation):

- anxiety attacks
- anxiety feeling
- adjustment disorder
- performance anxiety
- anxiety-related insomnia
- separation anxiety
- parental anxiety
- neurosis
- OCD (separate flag)
- PTSD (separate flag)
- phobias (separate flag)
- panic disorders (separate flag)

The following coded terms (from Docle or Pyefinch) have been used to identify records for inclusion:

- ADJUSTMENT DISORDER WITH ANXIETY

- ADJUSTMENT DISORDER WITH MIXED ANXIETY AND DEPRESSED MOOD

- ANXIETY

- ANXIETY - GENERALISED

- ANXIETY - PTSD

- ANXIETY - SOCIAL

- ANXIETY DISORDER

- ANXIETY DISORDER, SUBSTANCE INDUCED

- ANXIETY NEUROSIS

- ANXIETY PHOBIA

- ANXIETY WITH PANIC ATTACKS

- ANXIETY/DEPRESSION

- DEPRESSION/ANXIETY

- DEPRESSIVE ANXIETY DISORDER

- GAD

- GAD (GENERALISED ANXIETY DISORDER)

- GENERALISED ANXIETY DISORDER

- GENERALISED ANXIETY DISORDER (GAD)

- MIXED ANXIETY DEPRESSION

- MIXED ANXIETY/DEPRESSIVE DISORDER

- MIXED DEPRESSION ANXIETY

- NERVOUS ANXIETY

- NEUROTIC ANXIETY

- PHOBIC ANXIETY DISORDER

- SOCIAL ANXIETY DISORDER

- SOCIAL PHOBIA

- SUBSTANCE INDUCED ANXIETY DISORDER

Free text entries have been searched for texts strings matching the list of coded terms (above) as well as the following text strings:

- ANX

- AX

Records identified by a free text string alone are not automatically flagged, but are individually reviewed by a clinical coder to determine whether the text string actually refers to the condition indicated or is present in another context (eg, a search for 'cancer' may identify 'partner died from cancer'). Each record is flagged accordingly. Records indicating ‘suspected’, ‘query’, or ‘?’ are not flagged as the condition.

This condition flag was last updated in October 2019.

## Asthma

This flag indicates those records where Asthma (or a relevant synonym) is reported in MedicineInsight either as a coded condition (using a drop-down list in the CIS) or as a non-coded condition (free text) in one or more of the 'Diagnosis', 'Reason for visit' or 'Reason for prescription' fields.

The following coded terms (from Docle or Pyefinch) have been used to identify records for inclusion:

- ACUTE SEVERE ASTHMA

- ALLERGIC ASTHMA

- ALLERGY INDUCED ASTHMA

- ASPIRIN SENSITIVE ASTHMA

- ASTHMA

- ASTHMA - ALLERGY INDUCED

- ASTHMA - CHRONIC PERSISTENT

- ASTHMA - EXERCISE INDUCED

- ASTHMA - FREQUENT EPISODIC

- ASTHMA - INFECTIVE EXACERBATION

- ASTHMA - INFREQUENT EPISODIC

- ASTHMA - PRECIPITATED BY BACTERIAL INFECTION

- ASTHMA - PRECIPITATED BY VIRAL INFECTION

- ASTHMA ACTION PLAN

- ASTHMA ACTION PLAN PERFORMED

- ASTHMA ACTION PLAN PRINTED

- ASTHMA CARE PLAN

- ASTHMA CARE PLAN REVIEW

- ASTHMA CYCLE OF CARE

- ASTHMA EXACERBATION

- ASTHMA REVIEW

- ASTHMA, ALLERGIC

- ASTHMA, ALLERGY INDUCED

- ASTHMA, CHILDHOOD

- ASTHMA, EXERCISE INDUCED

- ASTHMA, FREQUENT EPISODIC

- ASTHMA, INFECTIVE EXACERBATION

- ASTHMA, INFREQUENT EPISODIC

- ASTHMA, OCCUPATIONAL

- ASTHMA, THUNDERSTORM

- BRONCHIAL ASTHMA

- CARE PLAN, ASTHMA

- CHECK UP, ASTHMA

- EXERCISE INDUCED ASTHMA

- EXERTIONAL ASTHMA

- FREQUENT EPISODIC ASTHMA

- INFECTIVE EXACERBATION OF ASTHMA

- INFREQUENT EPISODIC ASTHMA

- OCCUPATIONAL ASTHMA

- REVIEW - ASTHMA

- SAMTER'S TRIAD

- STATUS ASTHMATICUS

- THUNDERSTORM ASTHMA

- WHEEZY BRONCHITIS

Free text entries have been searched for texts strings matching the list of coded terms (above) as well as the following text strings:

- ASTH

- ASTMA

- SAMTER

Records identified by a free text string alone are not automatically flagged, but are individually reviewed by a clinical coder to determine whether the text string actually refers to the condition indicated or is present in another context (eg, a search for 'cancer' may identify ‘partner died from cancer’). Each record is flagged accordingly. Records indicating ‘suspected’, ‘query’, or ‘?’ are not flagged as the condition.

Note: Although ‘Wheezy bronchitis’ is not the most correct term at this time, it shares a code with Asthma and Bronchial Asthma and so has been included. It will however not be searched for as a free text term.

This condition flag was last updated in January 2019.

## Depression

This flag indicates those records where Depression (or a relevant synonym) is reported in MedicineInsight either as a coded condition (using a dropdown list in the CIS) or as a non-coded condition (free text) in one or more of the 'Diagnosis', 'Reason for visit' or 'Reason for prescription' fields.

The following coded terms (from Docle or Pyefinch) have been used to identify records for inclusion:

- ADJUSTMENT DISORDER (CHRONIC) WITH DEPRESSED AND ANXIOUS MOOD

- ADJUSTMENT DISORDER (CHRONIC) WITH DEPRESSED MOOD

- ADJUSTMENT DISORDER WITH DEPRESSED AND ANXIOUS MOOD

- ADJUSTMENT DISORDER WITH MIXED ANXIETY AND DEPRESSED MOOD

- ANXIETY/DEPRESSION

- CHRONIC ADJUSTMENT DISORDER WITH DEPRESSED AND ANXIOUS MOOD

- CHRONIC ADJUSTMENT DISORDER WITH DEPRESSED MOOD

- DEPRESSION

- DEPRESSION - ENDOGENOUS

- DEPRESSION - MINOR

- DEPRESSION - POST NATAL

- DEPRESSION - REACTIVE

- DEPRESSION - RECURRENT

- DEPRESSION - SUBSYNDROMAL

- DEPRESSION WITH MELANCHOLIC FEATURES

- DEPRESSION, ENDOGENOUS

- DEPRESSION, MELANCHOLIC

- DEPRESSION, NON MELANCHOLIC

- DEPRESSION, ORGANIC

- DEPRESSION, POSTNATAL

- DEPRESSION, PSYCHOTIC

- DEPRESSION, REACTIVE

- DEPRESSION/ANXIETY

- DEPRESSIVE ANXIETY DISORDER

- DEPRESSIVE EPISODE, MAJOR

- ENDOGENOUS DEPRESSION

- INSOMNIA - DEPRESSION-RELATED

- INVOLUTIONAL MELANCHOLIA

- MAJOR DEPRESSION

- MAJOR DEPRESSIVE EPISODE

- MELANCHOLIA

- MELANCHOLIA - INVOLUTIONAL

- MELANCHOLIC DEPRESSION

- MIXED ANXIETY DEPRESSION

- MIXED ANXIETY/DEPRESSIVE DISORDER

- MIXED DEPRESSION ANXIETY

- NEUROTIC DEPRESSION

- NON MELANCHOLIC DEPRESSION

- ORGANIC DEPRESSION

- POST NATAL DEPRESSION

- POSTNATAL DEPRESSION

- PSYCHOTIC DEPRESSION

- REACTIVE DEPRESSION

Free text entries have been searched for texts strings matching the list of coded terms (above) as well as the following text strings:

- DEPRES

Records identified by a free text string alone are not automatically flagged, but are individually reviewed by a clinical coder to determine whether the text string actually refers to the condition indicated or is present in another context (eg, a search for 'cancer' may identify 'partner died from cancer'). Each record is flagged accordingly. Records indicating ‘suspected’, ‘query’, or ‘?’ are not flagged as the condition.

Note: This condition flag was last updated in January 2019.

## Osteoporosis

This flag indicates those records where Osteoporosis (or a relevant synonym) is reported in MedicineInsight either as a coded condition (using a drop-down list in the CIS) or as a non-coded condition (free text) in one or more of the 'Diagnosis', 'Reason for visit' or 'Reason for prescription' fields.

The following coded terms (from Docle or Pyefinch) have been used to identify records for inclusion:

- OSTEOPOROSIS

- OSTEOPOROSIS - CORTICOSTEROID INDUCED

- OSTEOPOROSIS - NO FRACTURE

- OSTEOPOROSIS - PREVENTIVE CARE

- OSTEOPOROSIS WITH FRACTURE

- OSTEOPOROSIS, DISUSE

- OSTEOPOROSIS, STEROID INDUCED

- PATHOLOGICAL FRACTURE DUE TO OSTEOPOROSIS

- POST MENOPAUSAL OSTEOPOROSIS

- PREVENTIVE CARE - OSTEOPOROSIS

- STEROID INDUCED OSTEOPOROSIS

Free text entries have been searched for texts strings matching the list of coded terms (above) as well as the following text strings:

- OP

- OSTEOPOR

Records identified by a free text string alone are not automatically flagged, but are individually reviewed by a clinical coder to determine whether the text string actually refers to the condition indicated or is present in another context (eg, a search for 'cancer' may identify 'partner died from cancer'). Each record is flagged accordingly. Records indicating ‘suspected’, ‘query’, or ‘?’ are not flagged as the condition.

This condition flag was last updated in August 2018.

## Type 2 diabetes

This flag indicates those records where Diabetes mellitus, type 2 (or a relevant synonym) is reported in MedicineInsight either as a coded condition (using a drop-down list in the CIS) or as a non-coded condition (free text) in one or more of the 'Diagnosis', 'Reason for visit' or 'Reason for prescription' fields.

The following coded terms (from Docle or Pyefinch) have been used to identify records for inclusion:

- DIABETES MELLITUS - NIDDM

- DIABETES MELLITUS - TYPE II

- DIABETES MELLITUS, NIDDM

- DIABETES MELLITUS, TYPE 2

- DIABETES TYPE II REQUIRING INSULIN

- NIDDM

- NIDDM - REQUIRING INSULIN

- NIDDM (NON INSULIN DEPENDENT DIABETES MELLITUS)

- NON INSULIN DEPENDENT DIABETES MELLITUS

- T2DM

- TYPE 2 DIABETES MELLITUS

Free text entries have been searched for texts strings matching the list of coded terms (above) as well as the following text strings:

- DIABETES

- DIABETIC COMA - KETOACIDOTIC

- DIABETIC KETOACIDOTIC COMA

- T11

- TII

- Type 11

- Type 2

- Type II

- Type two

- Type2

Records identified by a free text string alone are not automatically flagged, but are individually reviewed by a clinical coder to determine whether the text string actually refers to the condition indicated or is present in another context (eg, a search for 'cancer' may identify 'partner died from cancer'). Each record is flagged accordingly. Records indicating ‘suspected’, ‘query’, or ‘?’ are not flagged as the condition.

Note: This condition flag was last updated in January 2019.
